# Opioids, microglia, and temporal lobe epilepsy

**DOI:** 10.3389/fneur.2023.1298489

**Published:** 2024-01-05

**Authors:** Lauren Marijke Lankhuijzen, Thomas Ridler

**Affiliations:** Hatherly Laboratories, Department of Clinical and Biomedical Sciences, University of Exeter Medical School, University of Exeter, Exeter, United Kingdom

**Keywords:** epilepsy, microglia, opioids, seizure, temporal lobe, inflammation, dynorphin

## Abstract

A lack of treatment options for temporal lobe epilepsy (TLE) demands an urgent quest for new therapies to recover neuronal damage and reduce seizures, potentially interrupting the neurotoxic cascades that fuel hyper-excitability. Endogenous opioids, along with their respective receptors, particularly dynorphin and kappa-opioid-receptor, present as attractive candidates for controlling neuronal excitability and therapeutics in epilepsy. We perform a critical review of the literature to evaluate the role of opioids in modulating microglial function and morphology in epilepsy. We find that, in accordance with anticonvulsant effects, acute opioid receptor activation has unique abilities to modulate microglial activation through toll-like 4 receptors, regulating downstream secretion of cytokines. Abnormal activation of microglia is a dominant feature of neuroinflammation, and inflammatory cytokines are found to aggravate TLE, inspiring the challenge to alter microglial activation by opioids to suppress seizures. We further evaluate how opioids can modulate microglial activation in epilepsy to enhance neuroprotection and reduce seizures. With controlled application, opioids may interrupt inflammatory cycles in epilepsy, to protect neuronal function and reduce seizures. Research on opioid-microglia interactions has important implications for epilepsy and healthcare approaches. However, preclinical research on opioid modulation of microglia supports a new therapeutic pathway for TLE.

## Introduction

Microglia act as the brain's primary, resident immune cells, working innately to defend threats to CNS homeostasis, safeguarding neuronal function (1–4). Insult to the CNS induces rapid transformation of microglia from a “resting” state, to a functioning, “activated” morphology, migrating to the site of injury to create an inflamed environment (2, 5, 6). In epilepsy, neuroinflammation can aggravate the excitability of neurons, decreasing the seizure threshold and increasing susceptibility to chronic seizures (7). Modulating microglial activation in favor of protecting neuronal function in epilepsy, may be possible by acute targeting of opioid receptors (8–10). The opioid system has been long implicated in the endogenous termination of seizures, with its unique ability to modulate neuronal excitability. Recently, a growing body of literature suggests that modulation of microglial activation by opioids represents a new pathway for therapeutic use in epilepsy, to promote anti-inflammatory properties of microglia and reduce seizure frequency and intensity (11–15).

Opioid receptors can be separated into three classic receptor types: the kappa opioid receptor (KOR), the delta opioid receptor (DOR), and the mu-opioid receptor (MOR), all Gi/o coupled receptors which can alter microglial reactivity through toll-like receptor 4 (TLR4) expression and regulation of the nuclear factor kappa B (NF-kB) pathway (8, 9). It has been shown that opioid receptor ligands can promote the polarization of activated microglia to their anti-inflammatory (often referred to as M2) phenotype, through inhibition of TLR4 and NF-kB (8). This supports a potentially new way to disrupt the self-perpetuating cycles of neuronal damage and inflammation that pro-inflammatory (often referred to as M1) microglia contribute to epilepsy (8, 9, 16). However, the complex interplay between opioids and neural circuits, alongside the need for a highly accurate drug application, makes it difficult to assess their true potential.

To evaluate, the current review will explore the role of opioids in modulating microglial activation in epilepsy, collating evidence to support the opioid system as a target for modulating microglia in TLE. The aim will be to (1) define the pro- and anti-epileptic properties of microglia, (2) determine the role of an endogenous opioid system in epilepsy and understand the scope for opioid receptor therapies in epilepsy, (3) determine the ability of opioids to modulate microglial function and morphology. To finally, evaluate the role of opioids in modulating microglial function and morphology in epilepsy, with a focus on potential future therapies.

To achieve the aims of this critical review, data was sourced from online databases including Google Scholar, PubMed, Science Direct, and Web of science with a particular focus on recent research [Example search terms included: (“opioids” OR “dynorphin” AND “microglia” OR “epilepsy”) and (“microglia” OR “opioids” AND “proconvulsant” OR “anticonvulsant” OR “epilepsy” OR “neuroprotective”)]. We focused specifically on research in temporal lobe epilepsy (TLE), as the most common condition, with other forms being beyond the scope of this review.

## Microglia in the epileptic brain

Under healthy CNS conditions, microglia maintain a ramified morphology, overseeing the health of neurons and synapses in the brain by fine interactions between their cell surface and neurons in their proximity (1–4). It is important to recognize that activation of microglia is complex beyond the binary states of “active” or “inactive” and their activity is highly dynamic – involving a multitude of morphological, genetic, and functional changes (1, 17, 18). Here, microglial “activation” is referred to as a change in gene expression and morphology with functional purposes. *In vivo* studies have shown that in response to damage or inflammation to the CNS, microglia transform from a ramified morphology with a small soma to an amoeboid state with an increased soma size (4, 5). This morphological transition also encompasses functional changes including the release of proinflammatory cytokines and chemokines (17, 19). Notably, within their reactivity, microglia can adopt different phenotypes consistent with multiple morphologies, leading to variable effects on the CNS. Traditionally, the extremes of these effects can be divided for simplicity into M1 or M2 microglial phenotypes (16, 18). M1 microglial polarization is associated with neurotoxic cascades and induced inflammation, caused by the release of pro-inflammatory cytokines and chemokines, whilst M2 microglia release anti-inflammatory cytokines and produce growth factors to resolve inflammation and neuronal damage (16, 18, 20). Proinflammatory cytokines such as tumor necrosis factor-alpha (TNFα) are capable of altering synaptic plasticity and neuron communication, having shown to modulate gamma-aminobutyric acid (GABA) and glutamatergic synaptic transmission, and thus cortical excitability (21, 22). Interleukins IL-1β, IL-2, IL-6, and interferon-gamma (IFN-γ) are also released by M1 microglia and can induce neuronal death and inflammation throughout the brain (18, 19). Contrastingly, M2 microglia secrete IL-4 and IL-13 cytokines and show neuroprotective properties in response to CNS inflammation, preserving neuronal function (18, 23, 24). While attribution of microglia into M1 and M2 phenotypes is likely an oversimplification, the general trend of these activation phenotypes has large implications in the epileptic brain, hence rendering their modulation as a key tool for treatment (8, 16, 25).

Inflammation is fundamental in the origin and the outcome of seizures in epilepsy (26–28). It is well understood that forms of acquired epilepsy can occur as a result of brain inflammation, and that pro-inflammatory cytokine molecules directly promote epileptic activity (26). For example, a relevant portion of temporal lobe epilepsy is related to autoimmune inflammatory processes such as limbic encephalitis (29). The events of a seizure trigger a cascade of complex immune signals, mediating inflammatory processes, clearing damaged or unfunctional cells, phagocytosis, and neuroprotection (28). Microglia remain upregulated around the area of insult for 4–5 weeks post-injury causing neuroinflammation (27, 28, 30). This is likely to progress epileptogenesis and aggravate neural injury by contributing to a decrease in seizure threshold (7, 31). Understanding microglial activation according to the timeline of epilepsy may provide a way to downregulate neuroinflammation by manipulating microglial polarization directly after insult (17). Microglia likely act as both anti-epileptic and pro-epileptic mediators in the brain (32). Earlier activation is suggested to be neuroprotective, whilst later stages of epileptogenesis cause increased inflammation and neurogenesis (17, 32, 33).

### Anti-epileptic role of microglia

It is clear that the inflammatory contributions of microglia to epilepsy are likely to exacerbate pathological conditions. However, therapeutic interventions targeting microglia must also consider their neuroprotective functions (32). A recent study (34) exploring the importance of microglia, adapted multiple genetic microglial ablation techniques, to examine their effects on kainic acid-induced seizures. Depletion of microglia caused increased seizure duration and intensity, higher mortality, and elevated neurodegeneration in the temporal lobe (34). These effects were ameliorated by replenishing microglia, suggesting that the simple ablation of microglia cells is an ineffective way to combat the pro-epileptic effects of microglia in epilepsy (34).

Microglia are known to phagocytose malfunctioning or dead progenitor cells in the dentate gyrus during neurogenesis (32, 35). Prolonged hyperexcitability of neurons, in the form of status epilepticus, causes a large increase in new-born cells of the dentate gyrus. Luo et al. (35) showed that microglia acted after status epilepticus to control levels of new-born cells by primary phagocytosis of viable cells, ultimately acting to protect neuronal function. This may explain why other studies (23, 36, 37) have reported a large increase in microglial phagocytosis post seizures but reiterates that this may not be a negative aspect of microglial response. Instead, increased phagocytosis of viable newborn cells acts to regulate dentate circuitry homeostasis and recover seizure damage (35). These results are reflected in further work, where microglia engage in neurogenesis of aberrant grown axons and dendrites of neurons after seizures (37). It has been reported that activation of microglial Toll-like receptor 9 regulated abnormal neurogenesis post-seizure in rodent seizure models and the absence of these receptors was responsible for increased seizure severity and seizure-induced cognitive decline (37). This represents how post-seizure microglia still function to maintain neural protection, and more research should investigate the phenotypes and stages where microglia exhibit protective behavior in neurogenesis (34). Studies tend to simplify stages in epilepsy to “before” and “after” seizures, yet the reality is more complex. Therefore, a timeline of microglial function and morphology in stages of epileptogenesis, as well as an account of synaptic maintenance and homeostasis in the epileptic brain would contribute valuable understanding (32).

### Pro-epileptic role of microglia

Microglia are expected to contribute toward epilepsy through their pro-inflammatory behavior in response to damage to the CNS (2, 17, 32, 38). Identifying the chemical response of microglia in epilepsy, Morin-Brureau et al. (27) used temporal lobe tissue obtained from human epileptic patient biopsies to measure microglial cytokine release post-induction of seizures with kainic acid. IL-1B, IL-6 and TNFα were all found at a higher levels to control subjects, with no seizures (27). Each of these are known to exert pro-convulsant behavior on glutamatergic and GABAergic signaling, to cause hyperexcitability, neuronal death and prolonged seizures (27, 39, 40). The study also highlighted an upregulated transcription factor in an element of the NF-kB pathway (27), furthering a pro-inflammatory response and promoting the idea of a therapeutic intervention to inhibit or suppress the upregulation of the NF-kB pathway (8, 25, 27). To gain a more comprehensive understanding of these cytokines in human epilepsy, it might be suggested that multiple cytokine measurements are taken at different times throughout the epileptogenesis timeline, from the initial CNS insult to the development of seizures.

Microglia likely indirectly progress neuronal damage after seizures by cytokine release but may also be directly implicated in neuronal damage through phagocytosis. Microglial phagocytosis has been considered an independent factor in microglial contributions to epilepsy and proposed as a potential target in novel therapeutics (36, 41–43). Microglial phagocytosis has shown to change in correlation with neuronal hyperactivity and inflammation (41, 42). One study (41) showed that inflammation *in vivo*, and hyperexcitability *in vitro*, under non-TLE conditions lead microglia cells to compensate for the increase in newly generated cells by accelerating phagocytosis [also seen in Luo et al. (35)]. However, interestingly, when applied to mouse models with TLE, and human TLE biopsy tissues, the balance between microglial phagocytosis and cell death was chronically lower than the experiments under non-TLE conditions. The decrease was hypothesized to be correlated to the increased microglial proinflammatory cytokine release. The increase in damaged cells was attributed to impaired microglial surveillance and cell recognition developed during the acute phase of epilepsy, suggesting that phagocytic efficiency had been damaged in models of TLE (41). A follow-up study (42) reported that when analyzing microglial phagocytosis in a TLE rodent model, phagocytosis was found to be localized to the granule cell layer and determined to be unrelated to seizures, yet potentially a result of local neuron connectivity in epilepsy. It was suggested that microglial phagocytosis may be an early marker of hippocampal damage in epilepsy and could be targeted in protecting neuronal function (42). The findings of microglial phagocytosis contributing to epileptogenic networks have been repeated in many recent studies (36, 41–43), however details concerning microglial phagocytic profiles or impact on the pathology of epilepsy disorder are still limited and more research is needed to understand non-inflammatory contributions to epilepsy, and how these may be targeted. Considering that impairment in phagocytosis was deemed correlated to proinflammatory cytokine release (41), it would be interesting to understand whether microglial phagocytosis impairment is related to different microglial morphologies and for example whether polarization to an M2 microglial phenotype would influence phagocytosis impairment. This could provide further reasoning for why altering microglial polarization may improve epilepsy.

Future clarification is needed to explain whether synaptic pruning of inhibitory synapses and phagocytosis performed by microglia, is correlated to increased vulnerability to epilepsy. We understand that certain behaviors associated with inflammation in the brain promote synaptic engulfment by microglia; however, its effect on epilepsy is not fully understood (17). The mechanism is likely to involve a contribution from astrocyte cells that are influenced by microglia (44), showing a need to identify these systems and further understand how we can disrupt their harmful properties.

### Targeting microglial properties in epilepsy

Microglia play an essential role in epilepsy, with their activation leading to significant consequences in the epileptic brain, both positive and negative (32). It should be noted that the activity of a seizure itself may cause damage to microglia, and more generally microglia's usual inflammatory response to injury seems to aggravate epilepsy pathology (7, 42). Benson et al. (16) measured the mRNA levels of M1-type properties, compared to M2-type properties of microglia in seizure-induced mice, using electro-encephalopathy measurements to examine seizure frequency. It was found that the events of a seizure abnormally upregulated M1 microglial activation, which in turn contributed to a more inflamed CNS, and likely caused more frequent and intense seizure activity. This describes a cycle, where the damage to microglia in epilepsy, in turn contributes to more frequent seizure activity. Therefore, promoting the M2-type microglia could attempt to counteract this inflammation, and interrupt cascades that follow M1-type activation, reducing seizure frequency and intensity.

Manipulating microglial phenotype could evidently be very influential in epilepsy treatment research (8, 25). However, some key areas must first be better understood for this to happen. If there are microglial injuries post seizure that disrupt their phagocytic role in neurogenesis, then it must be considered whether altering the microglial phenotype will resolve this (42, 43). Furthermore, astrocytes are also responsible for pro-inflammatory cytokine release in the epileptic brain, as they are activated in result of microglial activation, and then further enhance microglial activation once in an active state (22, 45). This complex interaction has been suggested to promote chronic microglial activation in temporal lobe epilepsy and thereby the overexcitement of neural circuitry (45). Such findings encourage future research to take a broader perspective and investigate microglia for their whole network and communications with other cells in the brain.

## Opioids in epilepsy

Around 70% of the seizures experienced in focal epilepsy can be localized to the temporal lobe, and in particular the hippocampus (9, 46). This aligns with the understanding that excessive glutamate release initiates seizure activity and neuronal death, as the hippocampus contains a high density of kainate, ionotropic glutamate receptors (9, 47, 48). Current epilepsy treatments use either antiseizure medication which generally act to block calcium or sodium channels in glutamate neurons, or take a surgical approach to sever connectivity within the area of interest. While the majority of patients attain seizure freedom, this enhanced inhibitory signaling comes at the detriment of patients experiencing nausea, depression, sedation, ataxia, and disappointing efficacy (9, 47). Shifting focus to drugs that could potentially modulate the excitability of the hippocampus with fewer side effects and more accuracy would provide a substantial benefit to treatment options. Drugs targeting opioid-receptor subtypes represent a clear target for such treatments and have displayed promising modulatory roles on a variety of seizures (9, 10, 13, 15).

The opioid system demonstrates strong control over hippocampal excitability, with contrasting influence over seizure expression, which is dependent on their receptor affinity, dosage or induction method (9). Endogenous opioids are synthesized directly within the temporal lobe, as well as a number of other areas in the brain, including afferents from the brainstem (49). Opioids have demonstrated direct interaction with principal hippocampal neurons through high receptor expression on GABAergic interneurons such as parvalbumin positive basket cells and somatostatin-expressing interneurons (50). Pathophysiological conditions in epilepsy can alter inhibitory activity of hippocampal interneurons and contribute to cell excitation and seizure vulnerability (51). Experimental silencing of hippocampal parvalbumin interneurons is sufficient to induce epilepsy in rodents (52), and thus the interaction of opioids with these cells is of significant interest in TLE pathology and treatment.

The three classic receptor types and endogenous peptides include the kappa opioid receptor (KOR) with endogenous opioid dynorphin; the delta opioid receptor (DOR) with enkephalin; and the mu opioid receptor (MOR) with endorphin. These are Gi/o coupled, 7- transmembrane domain proteins with high similarity in protein sequence, sharing a binding domain (53, 54). Opioids regulate neuronal excitation by acting on inhibitory pathways, and under the right conditions may deliver therapeutic anti-epileptic and anti-epileptogenic effects (8, 9, 55).

### Kappa opioid receptor and dynorphin

Dynorphins represent a class of endogenous opioid peptides, which target KOR, and emerge from the precursor protein prodynorphin (9, 10, 56). Dynorphin is abundantly expressed within the limbic system, particularly in mossy fibers of the hippocampus: the axons arising from dentate gyrus granule cells, cells that are structurally associated to chronic epilepsy (8, 9, 57, 58). In humans and rodents, the hippocampus has been found to internally release dynorphin during limbic seizure activity, with the activation of KOR providing negative feedback on excessive glutamate release in the hippocampus and helping to terminate seizure activity (10, 11). Human hippocampi specimens with TLE show a significant increase in prodynorphin mRNA expression in the dentate gyrus compared to control, highlighting a key role for dynorphin in endogenous termination of seizures (11). In addition, lower prodynorphin expression due to mutations of promoter regions has been associated with increased susceptibility to epilepsy in people with a family history of TLE (59, 60). Thus, using dynorphin to target KOR is of high pharmacological interest to control seizure activity in epilepsy (9).

Genetic polymorphisms in the prepro-dynorphin gene, cause reduced Dynorphin in humans, correlated to an increased susceptibility to developing epilepsy in human and mice models (13, 59, 60). Imaging studies show increased granular cell dispersion and neuronal loss in the hippocampus of prepro-dynorphin knockout mice and demonstrate a modulatory effect on neuronal excitation (10, 13). Loacker et al. (13) tested prodynorphin knockout mice, assessing their seizure threshold using a tail-vein infusion of a GABAa antagonist, and inducing seizures by injection of kainic acid. The prodynorphin knockout mice expectedly reported a decreased seizure threshold, a result which could be almost completely rescued by injection of a selective KOR agonist, effectively increasing the seizure threshold of mice back to wild type control subjects (13). These effects were isolated to KOR alone by employing a pre-treatment condition of KOR antagonists, which erased any positive effects. Interestingly, a delta receptor agonist was also used, which showed a decrease in seizure threshold for both transgenic and wild type mice, thus emphasizing the need for a highly specific KOR agonist for positive effects (13). This partly limits the study's implications for dynorphin, as it has reported some affinity for DOR (53), but to verify this, studies using dynorphin could test for DOR activation. If genetic polymorphisms in the prepro-dynorphin gene may predispose an individual to increased epilepsy risk, KOR agonism could be investigated to rescue these effects.

Studies have long supported the external activation of KORs to be effective in reducing the strength and tendency of limbic seizures (9, 13, 14, 55). KOR activation has been shown to limit neuronal excitability through its ability to reduce calcium uptake by blocking N-type calcium channels (10, 61, 62). These claims provoked clinical trials near the end of the 1990s, testing KORs agonists spiradoline and enadoline for therapeutic usage to reduce neuronal excitation. However, unfortunately they were failed for participants experiencing dysphoria side effects (10, 63). This halted all industrial research of KOR agonists as they were determined un-safe. More recently, Zangrandi et al. (64) saw that it was possible to pharmacologically separate the anticonvulsant effects achieved by targeting KOR from aversion side effects *in vivo*. This finding reignited the potential of KOR agonists as a therapeutic target for epilepsy, inciting more research to investigate whether this separation can be replicated in human TLE patients (10). With the mechanism of drug application of high priority, Agostinho et al. (55) demonstrated that by introduction of dynorphin through a release-on-demand gene therapy, drug-resistant TLE rodent models reported long term suppression of seizures, with minimized risk of side effects (55). Furthermore, the agonism of KOR during epileptogenesis has displayed increased neuronal survival and neuroprotective effects (14, 65). With development of new sensitive application methods, dynorphin-based clinical, anti-epileptic, treatments in humans show strong potential for being reintroduced to clinical trials.

There are many drugs that can be used to activate KOR and in general they have shown to suppress seizures in epilepsy and promote the survival of neurons in the amygdala and hippocampus of mice (13, 14, 55, 65). However, it should be considered that especially in genetic studies, results from rodents should not be directly extrapolated to humans. It has already been shown that seizures in rat models have higher up-regulation of neuropeptide Y from dentate granular cells, than dynorphin. Whilst, neuropeptide Y does not increase in dentate granular cells during human seizure activity (66). Since neuropeptide Y is homologous in humans and rodents, this indicates a different role of dynorphin and proposes a more significant inhibitory effect of dynorphin during seizure activity in humans (11, 67). Future studies should therefore aim to work with human participants with genetic variations in dynorphin precursor genes, for more application to therapeutics.

Gene therapy could see progression to human testing soon, as in rodent TLE models dynorphin-based gene therapy showed a complete termination of seizures within 2 months of therapy, whilst maintaining normal learning and memory (55). Within the same study, human TLE hippocampal biopsy slices showed successful reduction in epileptiform activity from activation *in vitro* (55). Using this approach, it would be of value to determine the possibility of pharmacologically avoiding dysphoric side effects and still providing a reduction in seizure frequency and severity *in vivo* (55). It should also be considered that alongside dominant expression on glutamatergic neurons, KOR is also expressed in some groups of GABAergic interneurons, pertaining to the possibility of specific conditions causing an excitatory effect (9, 68). This should be thoroughly assessed in future research using human participants, as adverse side effects will be limited through highly selective and locally restricted treatments.

Altogether, the current research strongly implies a protective role of dynorphin in regulating hippocampal excitability and epileptic seizures, with the KOR system presenting as a good target for anticonvulsant effects and future antiseizure medication (13, 14, 55, 65). The role of dynorphin is complex and has been implicated in increasing anxiety-like behaviors amongst other negative side effects, thus it must be handled in a delicate manner with thorough consideration for where in the brain it can act and to which receptors (56). The biological mechanisms behind dynorphins' effect remain unclear, and microglia cells may give answers to some of these questions. A recent study by Liu et al. (8) showed that the activation of KOR and Dynorphin promotes the M2 microglial phenotype, by inhibition of the TLR4/NF-κB pathway. This perhaps explains partially why dynorphin has an anticonvulsive effect in the hippocampus, as increased M2 microglial polarization promotes anti-inflammatory and neuroprotective effects in epilepsy (24, 25).

### Delta opioid receptor and enkephalins

Enkephalins are endogenous opioid peptides targeting DOR. Structurally, they can be separated into met-enkephalin and leu-enkephalin peptides, produced from the proenkephalin precursor protein (9, 69, 70). Enkephalins are strongly expressed in the basal ganglia, comparatively much lower to the hippocampus; however their expression in the hippocampus increases after the induction of seizures in rat models (9, 69). Rat models show generally limited enkephalin expression in characteristic granule cells, and low levels of expression in pyramidal cells of the CA3 region (67). Whereas, human hippocampi show enkephalin immunoreactivity in several granule cells and pyramidal cells of the hippocampus, thus perhaps indicating a more significant role in humans than in rodents (9, 71).

The placement of enkephalins in the human brain make them suitable candidates for targeting in epilepsy; however, there is still debate surrounding their pro-epileptic effects. Enkephalins prioritize binding to DOR, although also have a high binding affinity for MOR, similar to morphine (54, 72). The activation of both DOR and MOR uses the reduction of GABAergic input, thereby creating disinhibition in the hippocampus and promoting synaptic plasticity (24). This has been linked to proconvulsive effects in the brain and increased vulnerability to epileptic seizures (9, 72). Research has therefore been more inclined to antagonize DOR under epileptic conditions, with several studies reporting that DOR antagonism is able to block seizure activity through regulation of GABAergic transmission (9, 73). Addressing concerns of dependency, one study also reported that DOR antagonism does not cause behavioral signs of withdrawal in morphine-dependant rodents, yet still reduced epileptic activity (74). Although, the placement of enkephalins and DOR in the brain does support its role in regulating inhibitory transmissions, its antagonism is still far from successful due to the many adverse side effects such as depression and pain (72). One other large drawback with targeting DOR is that the inhibition of GABAergic interneurons may modulate excitatory signaling, but it also may weaken the synchronization and control of the hippocampal brain region, giving limited control over its effects (9). Nonetheless, with growing pharmacological advances, DOR should not be fully discounted for epilepsy research. Enkephalins and DOR play a role in modulating seizure activity endogenously, and drugs exploiting the neuroprotective effect of DOR with high specificity, could be of significant interest to the future of epilepsy treatment.

A potential mechanism behind enkephalins endogenous control of seizures, may be explained by their effect on microglia. A recent study found examples of how enkephalin peptides function on microglial polarization, namely methionine enkephalin, which pushed microglia to an M1 phenotype, with high levels of M1 markers, including TNF-a, CD86, CD40, IL-12, with no effect on M2 markers (75). This alludes to the possibility of enkephalins promoting the inflammatory microglial activation in the hippocampus and facilitating seizures (75). This supports the proconvulsant role of some enkephalins and may explain the observed hyperexcitability of hippocampi from activation of DOR (72). It would be of relevant interest to investigate whether antagonism of DOR could depict opposite “anti-inflammatory” effects in microglia and influence the polarization of microglia to an M2 phenotype, as this may give insight into the impact of targeting DOR for therapeutic purposes.

### Mu-opioid receptor and endorphins

Endorphins are the last group of endogenous peptides, which come in the form of α-, β-, γ- and δ-endorphins, and are derived from proopiomelanocortin (POMC) (9, 70). POMC is abundantly expressed in the dentate gyrus, and MOR have been identified by autoradiography in all regions of the hippocampus, however presence of β-endorphin in the hippocampus is still speculated (9). Activation of MOR causes modulated synaptic plasticity in the *Cornu ammonis regio superior* (CA1) of the hippocampus and disruption of CA1 neuronal activity (70). There is some evidence to suggest that MOR could be altered in TLE patients, promoting their relevance in future clinical intervention of epilepsy (76–79). One study found that onset of epileptic seizures at older age was associated with increased MOR binding and mu agonist activation, whilst longer-term chronic epilepsy saw a correlation to reduced MOR binding (72). This may be explained by a desensitization of MOR binding after recurring activation of receptors from seizures and suggests MOR expression may exert pro-convulsant effects in epilepsy.

Several studies (76–79) have reported increased morphological alterations and binding of MOR upon induction of seizures in animal models and PET imaging of TLE patients. Skyers et al. (78) showed that seizures caused an increase in terminals that expressed MOR in the inner molecular layer of rats, suggesting increased MOR activation after seizures (78). This potentially acts to disrupt restorative mechanisms, with MOR activation potentially inducing proconvulsant effects (80). In human, adult TLE patients, upregulated MOR binding also occurs, but seems limited to the temporal neocortex (76, 77).

Tramadol is an opioid peptide widely used in analgesia, with highest affinity for activating MOR and shows dose-dependent effects on convulsive activity (81, 82). One study found it displayed an anticonvulsive effect on rodent seizure models, when administrated within the analgesic range (82). However, other dosages showed opposite effect, causing convulsive seizure activity at higher doses (79). Also reported in human tramadol overdoses, seizures were highly associated with increased tramadol intoxication (82–84). There have also been case studies of a patient on low-dose tramadol for pain relief, developing chronic seizures from general low-dose tramadol (81). Therefore, the interplay of MOR in seizures is of high clinical importance to pain relief therapies and should be further investigated. It is likely that the many discrepancies in this field of research come from different induction methods, dosages and models used, highlighting no clear effect of MOR agonists, and thus currently not preferred targets for clinical intervention of epilepsy.

The explanation of MOR's effects are complex, however it remains possible that MOR promote a proinflammatory microglial phenotype (85). One study looking at treatment options for opioid dependence found that morphine mediated the proinflammatory M1 phenotype of activated microglia, through activation of MOR mediated signaling pathways (85). Furthermore, activating MOR has been associated to LPS-induced NF-kB activation, showing polarization of M1 polarized microglia (86). This could suggest that modulating MOR signaling by selective inhibition of downstream signaling pathways could influence the polarization phenotype of microglia and perhaps play a neuroprotective role in epilepsy. However, research on this is scarce and due to the high MOR receptor density it will likely take time for drugs to become specific enough to help our understanding of how MOR might play a regulatory role in epilepsy.

### Causative relation to microglial activation and therapeutic potential

Consistently, the anticonvulsant or proconvulsant role of each opioid receptor aligns with the proinflammatory or anti-inflammatory phenotype of microglia – implying a causative interaction between the activity of microglia and convulsive behaviors. Several studies (8, 75, 85) have shown that activation of opioid receptors, in some cases with their endogenous peptides, can modulate the role of microglia cells, which could have therapeutic implications for many CNS diseases, especially epilepsy.

As research currently stands, KOR and dynorphin show promise as a target for therapeutic treatment in epilepsy. A recent, literature review published by Zangrandi et al. (10) highlighted significant potential for a KOR dynorphin target in treating epilepsy in the future, with a further need for investigating long-term overexpression of KOR receptors (10). In some cases, opioid receptors can remain activated and cause lasting impact on neurotransmission, even after agonism is halted or receptors are antagonized. Contrastingly other opioid receptors are dependent on persistent agonism to function, and whilst few become desensitized, others do not (70). Therefore, when targeting any opioid receptors, thorough consideration must be taken for their long-term activation and to avoid adverse effects, high specificity should be employed in drug delivery.

## Microglia and the opiate system

**Figure 1:**
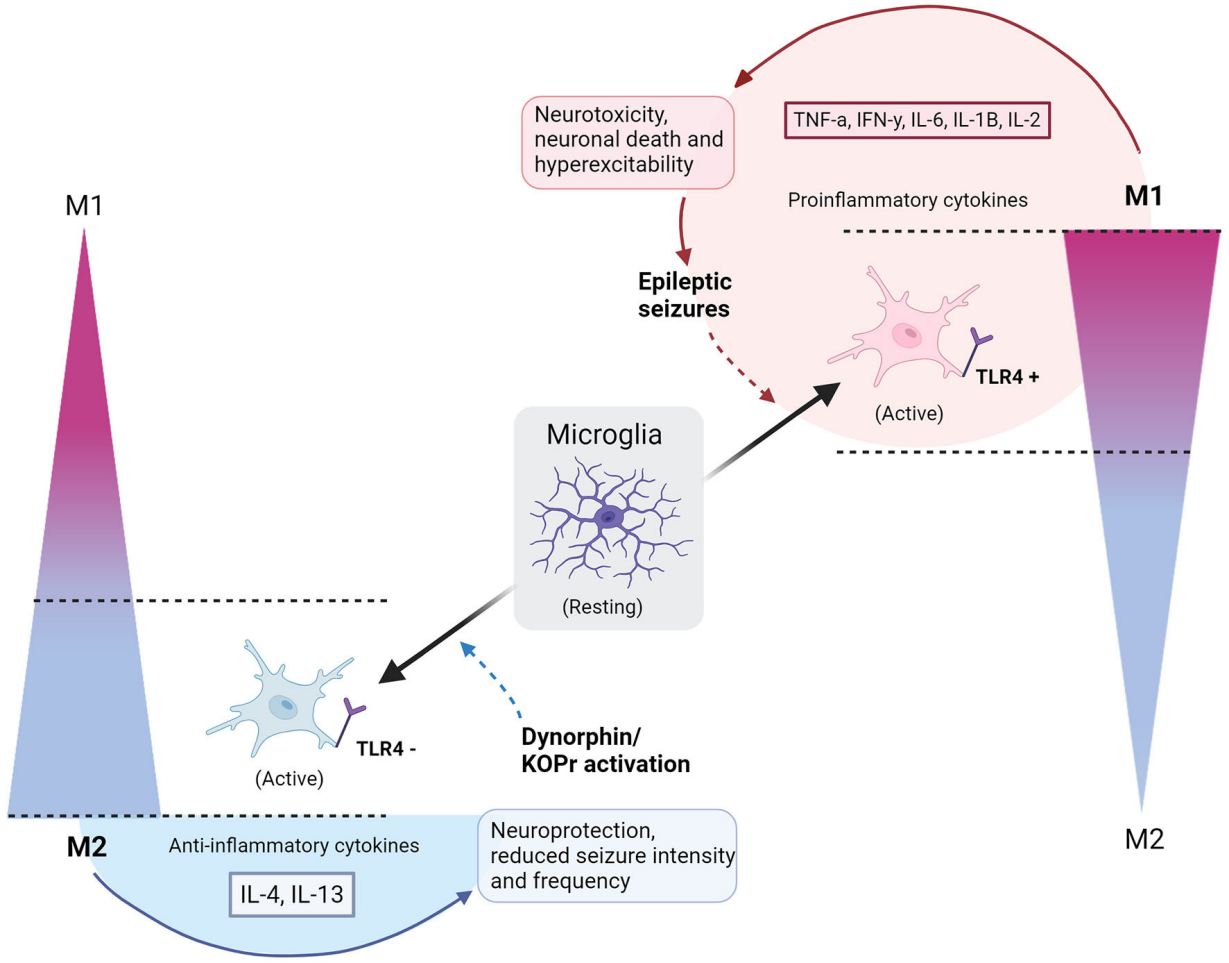
Polarization of microglia to M1 or M2 phenotypes with consequences of epileptic seizures or dynorphin/KOR activation. Resting microglia polarize to a simplified scale of activated M1 or M2 microglia phenotypes, indicated by gradients on the right and left sides of the diagram. Full, gradient arrows, indicate activation of microglia to polarized states, named by the direction of gradient (M1 or M2). “+” shows activation of TLR4, whilst “−” refers to an inhibition of TLR4. Full arrow leading from M2 microglia encompasses secretion of IL-4 and IL-13 anti-inflammatory cytokines and causes neuroprotection and reduced seizure intensity/frequency. Full arrow leading from M1 microglia encompasses secretion of TNFa, IFNy, IL-6, IL-1B, IL-2 pro-inflammatory cytokines to cause neurotoxicity, neuronal death and hyperexcitability, leading to epileptic seizures. Dashed arrow indicates a contribution to an altered polarized state – dynorphin/KOR promotes polarization of M2, whilst epileptic seizures upregulate M1 polarization. Circle by M1 polarization indicates a cycle of neurotoxic events. Semi-circle by M2 shows that neuroprotection is dependent on external application of dynorphin/KOR action. Created with BioRender.com.

**Figure 2:**
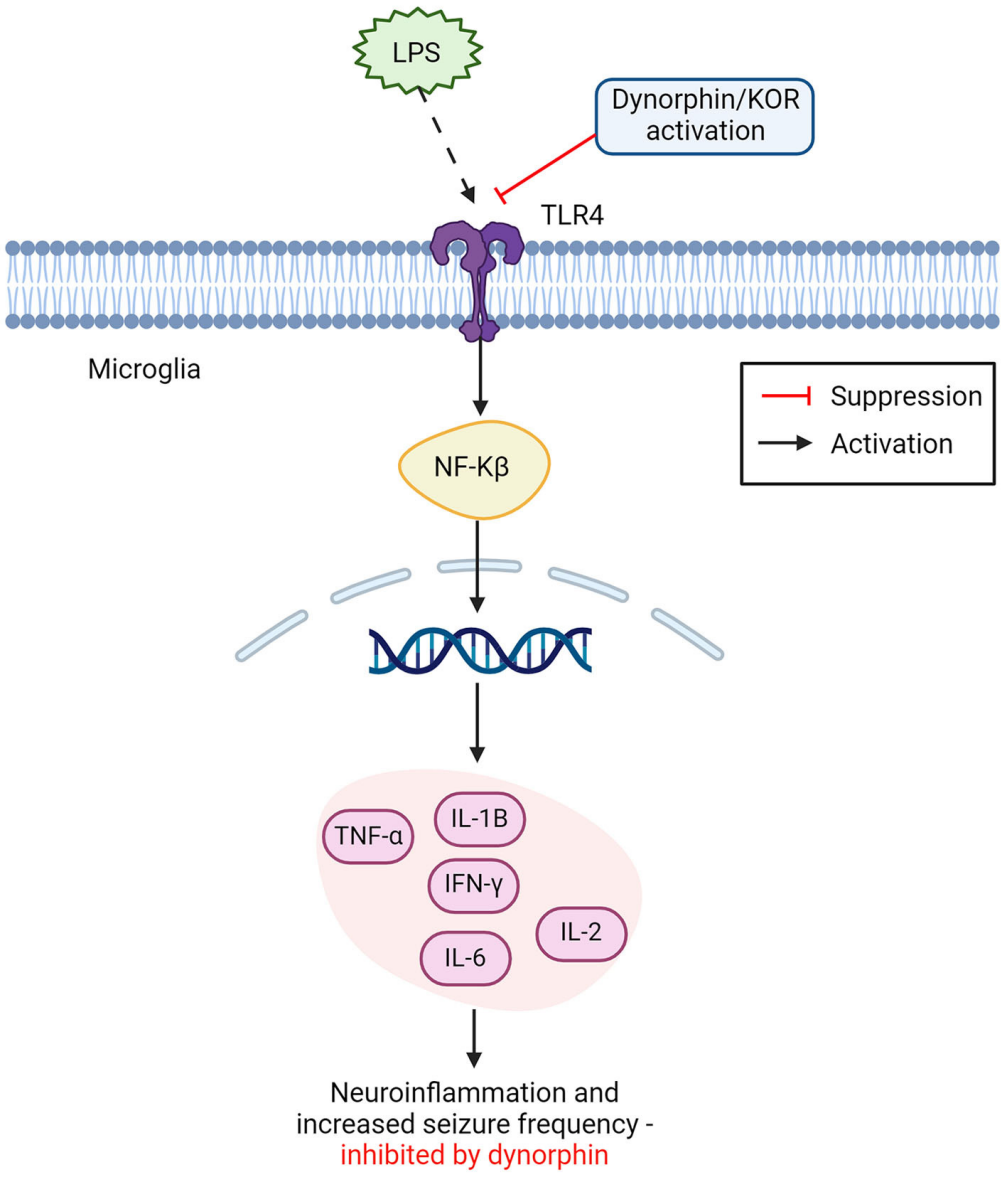
Opioid dynorphin causes suppression of microglial TLR4/NFkB inflammatory pathway. Dynorphin and or KOR activation shows competitive inhibition of LPS binding to TLR4 on microglia cells. Leading to suppression of the expected inflammatory activation from NFkB. LPS binding with TLR4 leads to NFkB activation in the nucleus, releasing proinflammatory cytokines and causing increased neuroinflammation and seizure frequency. This inflammatory pathway can be suppressed by dynorphin and or the activation of KOR (TNF-α, tumor necrosis factor alpha; IL-1β, interleukin 1β; IFN-y, interferon gamma; IL-2, interleukin 2; IL-6, interleukin 6; NFkB, nuclear factor kappa B; LPS, lipopolysaccharides; KOR, kappa opioid receptor). Created with BioRender.com.

Research into opioid-microglia interactions has traditionally drawn interest in fields of reward, addiction, and analgesia, however, using opioids to manipulate microglial polarization may be beneficial in reducing inflammation in a variety of CNS diseases (19, 25). One of the biggest advantages that opioids have in controlling microglial reactivity, is their activity on TLR4. TLR4 is located primarily on microglia cells and can directly modulate microglial function, causing downstream modulation of neuroinflammation (19, 87). Binding of lipopolysaccharides (LPS) to TLR4, triggers M1 microglial neuroinflammation by initiating activity of the NF-kB pathway, directly correlated to the expression of numerous proinflammatory cytokines with damaging effects on the brain (18, 19, 88–90). This process can be avoided by purposefully inducing polarization of M2 microglia, by inhibition of TLR4 and therefore the NFkB pathway, promoting neuroprotection (process outlined in Figure 1) (8, 24, 25). Opioids have high binding affinity to TLR4 and have shown to successfully promote M2 polarization through TLR4 inhibition, leading to reduced proinflammatory cytokine production (8, 25). Displayed in Figure 2, this is done by suppressing the binding of LPS to TLR4, and thereby suppressing the TLR4/NFkB proinflammatory pathway (18, 25). Counteracting the binding of LPS with opioids, such as dynorphin, to reduce neuroinflammation, may therefore have potential in treatment of inflammatory related brain disorders such as epilepsy.

As previously determined, opioid activity on microglia cells is highly dependent on the class of opioid receptors that is targeted. Morphine and tramadol are opiate drugs used frequently in pain relief, and their affinity for DOR and MOR, cause unique effects on microglia (20, 91). In mice, exposure to morphine causes a dramatic increase of proinflammatory microglial activation compared to vehicle drugs, resulting in neurotoxic cascades of upregulated M1 microglia (20, 92). This could potentially explain why, clinically, high doses of intravenous morphine can induce seizures in human epilepsy patients that are undergoing pain-relief (93). Similarly high doses of tramadol have shown to induce seizures in some patients, perhaps relevant to their action on MOR (81–83). This is also seen in addiction, where increased usage of morphine, tramadol, or similar substances such as fentanyl can cause repeated seizures in users, sometimes fatal (94). While in the clinic, these situations may be rather rare, and in most cases such drugs are clearly safe in the hands of anaesthesiologists, more awareness of the role of opioid receptors and microglial effects on hippocampal excitability could improve medical usage in the long term. Perhaps this awareness would, however, help to progress finding a safer way to target these receptors without severe neuroinflammatory effects.

A recent study by Mali et al. (25) shows that opioid agonists can change microglial morphology, acting to reduce neuroinflammation. Opioid activation caused reduced nitric oxide production in microglia cells (free radicals produced by activated microglia), accompanied with reduced inflammation in the CNS (25). This suggests that targeting opioid receptors was an accurate way to suppress neuroinflammation and stabilize an M2 polarized phenotype (25). Interestingly, this result was found using an agonist for each of the three opioid receptors, in contrast to some previous research stating that MOR and DOR cause M1 polarization *in vivo* (75, 85, 86). Therefore, further studies are needed to clarify these findings *in vivo*, with the influence of other neural circuits in the brain. However, this remains a very promising finding for the progression of treating CNS diseases associated with inflammation.

### Receptors on microglia, targeted by opioids, play a role in hippocampal excitability

Some receptors located on microglia cells can facilitate neuronal excitability through innate immune processes, offering a way to modulate seizure activity (17, 32, 37, 87). As previously discussed, TLR4 is a key receptor in mediating neuroinflammation through its expression on microglia cells (19, 88, 89). Pharmacological inhibition of TLR4 on microglia has been suggested to reduce pro-inflammatory microglial activation in seizures, and opioids show strong regulation of TLR4 (8, 19, 95).

It was recently shown that external inhibition of TLR4 located on microglia, displayed a neuroprotective effect in epileptic mice models, by promoting microglial polarization from an M1 phenotype, to an M2, anti-inflammatory phenotype (87). This result could not fully be explained, as the exact inhibitory role of TLR4 in neuronal excitability is still not fully understood and authors advised that further research should consider exploring TLR4 in therapeutic pathways for epilepsy (87). One possible explanation may be found in the interaction of TLR4 with the opioid system. A different study (96) found that the endogenous opioid dynorphin exhibits a competitive inhibitory effect on the TLR4 signaling pathway, potentially acting as an antagonist to TLR4 by suppressing the binding of LPS. As seen in Figure 2, the inhibition of TLR4 receptors was suspected to cause downstream suppression of the NF-kB pathway, responsible for the release of harmful pro-inflammatory cytokines (86, 96). This corresponds with findings that endogenous dynorphin causes increased polarization toward the M2 phenotype, as suppression of LPS binding to TLR4 reduces polarization to M1 microglia, further explaining the neuroinflammatory role of TLR4 and supporting dynorphins' anti-inflammatory effect on the epileptic brain (8). This approach to understanding dynorphins' anti-epileptic effects presents a strong argument for their use in therapeutic intervention of epilepsy. As it is already a process that occurs naturally within the brain to modulate the immune response, and its enhancement could give a long-term suppression of seizures with limited adverse side-effects (8, 10, 96).

The model in Figure 1 depicts the cycle of (1) epileptic seizures (2) upregulated M1 microglia (3) TLR4 activation (4) hyperexcitability and neurotoxicity, leading to repetition of the process. Introducing dynorphin might present a way to counteract this self-perpetuating neurotoxic cycle in epilepsy patients, promoting neuroprotection and decreasing seizures (8, 16). To confirm this as a viable treatment option, studies must include data on human epileptic hippocampal tissue that undergo treatment with dynorphin at safe amounts, performing quantitative analysis of cytokine levels and biomarkers for M1 and M2 positive microglia cells. This will reveal whether, in human tissue, the neuroprotective observations are mirrored to those in rodents. Furthermore, the method of applying dynorphin is highly implicated in its effects, with certain forms of administration found to cause tolerance and increase anxiety behaviors (56). The effects seen by agonists acting on KOR differ depending on intracerebroventricular or subcutaneous administration (10, 55). A dynorphin “release on demand” gene therapy has been found to give long-term suppression of seizures in epileptic mouse models, without adverse effects of anxiety, memory impairment, or tolerance, highlighting how such effects can be avoided with considered application methods (55). Lastly, it remains essential to not discount the potential benefits of anti-epileptic microglial properties. As already discussed, there are protective roles observed by microglia in the epileptic brain, and if attempting to modulate microglial polarization with dynorphin, it should be ensured that the neuroprotective roles of M1 phenotypes are taken into consideration (32).

Other receptors that are directly targeted by opioids include the MOR, located on microglial cells (97, 98). This relationship has been mainly explored for its role in opioid tolerance and withdrawal, as opioids targeting MOR cause microglial activation which dysregulates reward circuitry in mesolimbic areas (97). Due to reward pathway disruption, along with the potentially pro-inflammatory effects of targeting MOR, their receptors are less strong candidates for mediating microglial activation as a treatment in epilepsy (9, 19, 97). However, drugs with higher binding specificity for MOR give the potential to avoid disruption of reward circuitry and pro-epileptic effects in the future should not be fully dismissed.

### Opioids cause functional changes in microglia which influence epileptogenesis

Emerging literature suggests high heterogeneity in the morphology and function of microglia, depending on their location in the brain (2, 4, 18). Microglia display a significant change in the face of neural injury, and infusion of certain drugs, or endogenous compounds which have further implications on their functions (2–4). Considering the importance of their plasticity and the consequences of their activation, the changes that opioids cause for microglia are very relevant to their therapeutic potential in epilepsy.

**Figure 3:**
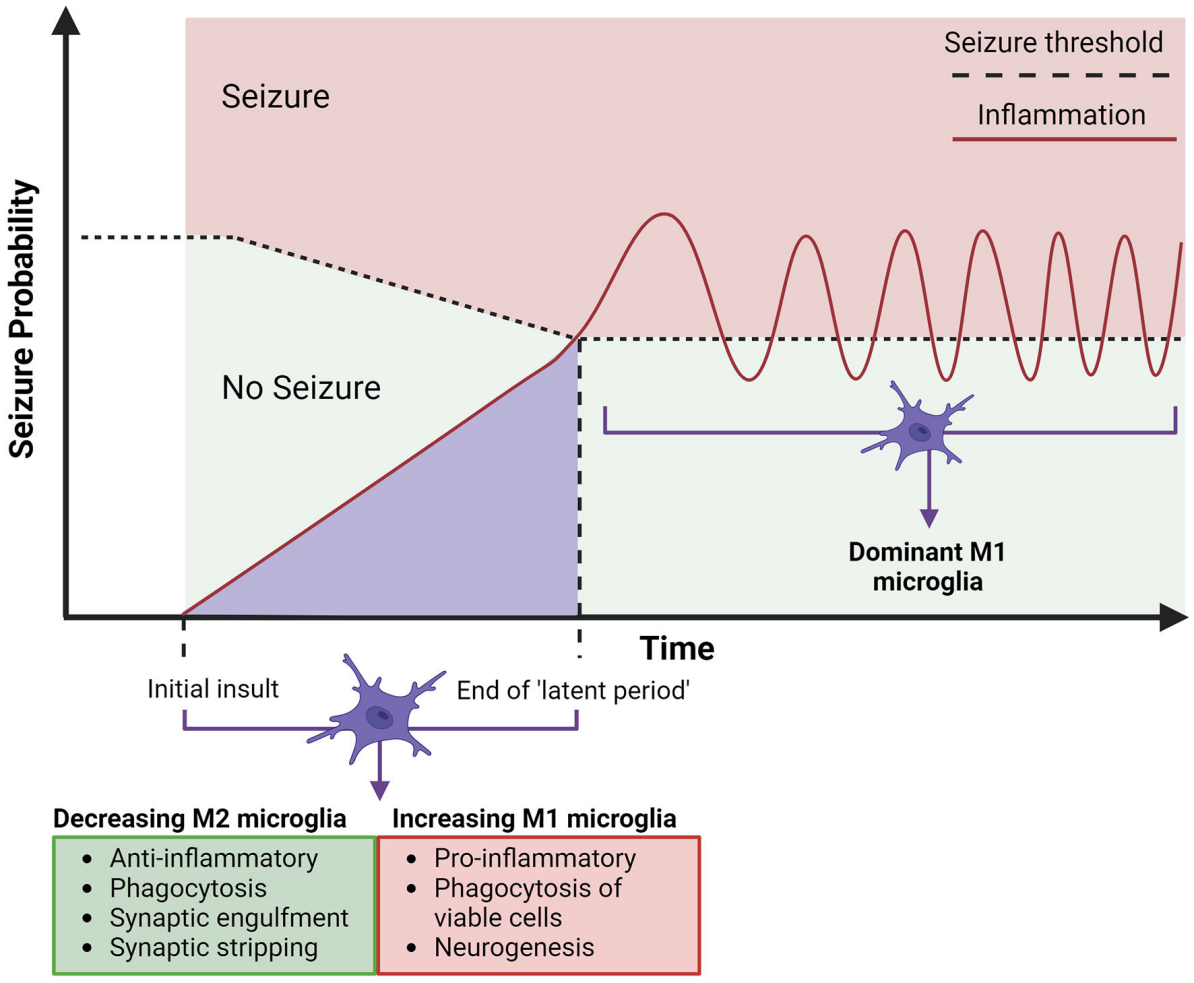
Timeline for the expected involvement of microglia in acquired temporal lobe epilepsy. Acquired epilepsy in the temporal lobes can be summarized to an initial insult, which leads to epileptogenesis, building up to a first seizure, which is followed by chronic and spontaneous seizures (epilepsy). The initial insult of CNS damage (represented by the lighter purple area) will instigate microglial activation, both anti-epileptic (M2) (arrow indicates these properties decreasing) and pro-epileptic (M1) (arrow indicates these properties increasing), alongside an increasing hyperexcitability of neurons, attributed to neuroinflammation from M1 polarized microglia. This may be sufficient to cause epilepsy, consistently lowering the seizure threshold (depicted by horizontal dotted line) by increasing neuronal excitation, leading to the eventual end of the “latent period” (period since initial insult - can be days, weeks, months, or years). The first seizure will trigger further neurotoxic cascades by upregulated M1 polarized microglia and hyper-ramified morphology, which will in turn continue chronic M1 microglial activation and further neuroinflammation, contributing to development of acquired epilepsy (33). Created with BioRender.com.

The usual “ramified” shape of microglia, has been found to turn hyper-ramified meaning larger and more “bushy', after the events of a seizure in kainic acid rat models (99, 100). Some studies (30, 101, 102) have shown that this can last several weeks after the seizure and cause a general hyper-ramified morphology of microglia in people with TLE. This is damaging to the brain, as the morphology is associated with proinflammatory functional changes such as cytokine production, faulty phagocytosis, further microglial activation, and upregulation of M1-type polarization cells (6, 17, 103). Figure 3 proposes a model for the progression of acquired TLE, where the increase of M1 microglia post-brain insult (such as head trauma, seizure, stroke, etc.) can contribute to epileptogenesis and cause the development of epilepsy. The model outlines how increased neuroinflammation contributes to a lowering seizure threshold, two factors that if progress will result in a seizure and the development of acquired epilepsy. Integrating what is proposed in Figure 1, intervention by modulating microglia with dynorphin/KOR activation in early stages of epileptogenesis to lower inflammation and to avoid secondary insult, may prevent the progression to epilepsy. Some M1-type microglia changes that occur after initial insult has been possible to ameliorate through the activation of opioid receptors, with reduced neuroinflammation showing protective potential in epilepsy (8, 18, 24, 25). Activation of KOR has been shown to increase neuronal survival, reduce inflammation and alter calcium channel activity to reduce excitability in the hippocampus, after injection of kainic acid in rodents (10, 61, 65). With the possibility to oppose progressing epileptogenesis, targeting KOR may also be effective in stages of chronic seizures. The upregulated M1 expression in epilepsy persists into the chronic epileptic stages of epilepsy where they are most likely to cause another seizure with stronger intensity (16). Activation of opioid receptors and specifically KOR has demonstrated a way to prevent excessive M1 expression and support anti-inflammatory cytokine release (IL-4, IL13) seen as neuroprotective in TLE. Having determined that opioids can suppress a neuroinflammatory response in the brain by promoting M2 expression, this should be further explored in treatments for epilepsy.

Few studies have investigated the influence of opioids on phagocytosis as this remains another major responsibility of microglia, relevant in epilepsy (25, 41, 42). As previously discussed, Mali et al. (25) demonstrate that opioid agonists can suppress neuroinflammation through M2 polarization. This further adds to the potential neuroprotective effect of opioids in epilepsy, as neuroinflammation causes excessive phagocytosis, which is suggested to advance epileptogenesis (17, 36, 42). However, as also discussed previously, increased phagocytosis after seizures could also be seen as a neuroprotective mechanism to maintain dendritic homeostasis, in compensation for increased cell generation after a seizure (35). Therefore, the protective effects of opioids on microglial phagocytosis are not fully clear and require studies to accurately determine whether reduced phagocytosis after seizures has neuroprotective benefits or disrupts homeostasis. This could be achieved by using detection antibodies to assess viable and damaged neurons before and after seizure activity with opioids induced M2 polarization and without, *in-vivo*. However, although M1/M2 polarization can indicate microglia activation, research should be vigilant of employing a simplified view of these complex processes, as categorization of microglia by polarization phenotype may not be the most accurate approach (104, 105). In any case, focus should be concentrated on the inflammatory and phagocytosis behavior of microglia.

### Therapeutic implications and direction of future research

It is clear that reducing neuroinflammation is likely to ameliorate neuronal damage and improve seizure outcomes in TLE patients (7, 8, 25, 31). Strategies are currently being developed on the most efficient ways to do this, with new drugs attempting to disable pro-inflammatory cytokines, target TLR4 or NF-kB signaling pathways, and block voltage-gated channels (2, 3, 18). However, many of these activities relate back to the actions of microglia cells, promoting them as a target for reducing neuroinflammation and potentially managing epileptic seizures (7, 32, 102). As the depletion of microglia has been deemed to aggravate seizures, the most effective way to manage their properties is by altering microglial polarization toward an M2 phenotype (34). In the course of this review, it has been highlighted that M1 polarized microglia might be increased in epilepsy, responsible for a constant cycle of inflammation, contributing to chronic seizures (16, 17). Leading to the hypothesis that interrupting pro-inflammatory M1 cascades could alleviate the severity of seizures in epilepsy. Furthermore, it seems that promoting M2 polarized microglia might have other benefits alongside inflammation, including phagocytosis and synaptic maintenance – deemed impaired in epileptic brain tissue (25, 41, 42). More research is needed to clarify M2 microglial roles in non-inflammatory processes and the most efficient way to achieve microglial polarization. This should also be explored for other CNS disorders, where neuroinflammation is destructive (2). Moreover, research on opioid effects on microglia is also relevant in other healthcare practices. Opioids are commonly used as medicinal pain relief, anesthetics, and a common substance in addiction, thus their effect on neuroinflammation should be more thoroughly investigated (85, 93, 97).

The opioid system has been shown to control microglial activation through receptors including TLR4, by exerting neuroprotective, anti-inflammatory effects under certain conditions (8–10, 19). KOR activation by dynorphin has already been introduced by adenno-associate virus (AAV) vector-based gene therapy in accurate delivery to TLE rodent models, demonstrating suppression of seizures within days (55). This was found to not disturb the cognitive ability, withdrawal/tolerance, or anxiety behaviors of rodents (55). Due to the diverse effects of opioids and their involvement in reward pathways, their application is critical and requires a high degree of accuracy (19, 97). Currently, there are several approved AAV vector-based gene therapies, with many still in clinical trials (106). As a growing area of research, this could be a promising way of delivering restricted treatment for epilepsy. However, other drug vehicles are also gaining interest, nanoparticles potentially presenting an easier way to deliver opioids, and should also be explored in this field (107). As previously discussed, there is likely to be a genetic element to epilepsy, which may involve opioid receptors, as mutations causing lower prodynorphin expression were demonstrated to correlate with increased susceptibility to epilepsy in people with a family history of TLE (59, 60). Future research could employ bioinformatics, to further an understanding of the genetic variation of endogenous opioids in TLE (108). There remain natural ways to increase endogenous dynorphin levels in the brain such as exercise, and some literature (109, 110) have supported gentle exercise such as aerobics to significantly decrease the number of seizures in some people. This has not been directly correlated to dynorphin and is likely more complex than decreasing seizures due to increased dynorphin levels from exercise, however, it engages in the challenge to naturally reduce seizures.

## Conclusions

The current treatment options for epilepsy concentrate largely on targeting neuronal excitability and have thus far been unsatisfactory in treating the chronic neuronal hyperexcitability experienced in TLE. The emerging role of abnormal microglial activation in fuelling inflammation, as well as reducing seizure threshold, presents an exciting alternative target for intervention in the early stages of epileptogenesis, or combating neuroinflammation in later stages of chronic epilepsy. Interrupting the neurotoxic cascades of upregulated M1 microglia, by modulated activation using dynorphin and KOR shows the potential to protect neuronal function and reduce seizure intensity, and frequency. However, for this to be successful, a pharmacological intervention targeting microglia must employ high levels of accuracy within the brain, and all pro-epileptic and anti-epileptic roles of microglia must be fully evaluated under treatment conditions. KOR activation achieves credible control of neuronal excitability and predictable anticonvulsant and anti-inflammatory effects under regulated conditions, in accordance with modulating microglia to an M2 phenotype. With the current understanding of how microglia, epilepsy, and opioids interact separately, as well as together, there is a promise for novel therapies applying opioids to modulate microglial activation in epilepsy.

## Author contributions

LL: Conceptualization, Investigation, Visualization, Writing—original draft. TR: Project administration, Supervision, Writing—review & editing.
